# Reinforcement Learning-Based Switching Event-Triggered Control for MWMR Under Asymmetric Actuator Saturation

**DOI:** 10.3390/s26216900

**Published:** 2026-10-31

**Authors:** Jie Bai, Yonghua Peng, Yunfa Wu

**Affiliations:** 1 School of Mechanical and Electrical Engineering, Guangdong Open University, Guangzhou 510091, China; jbai@gdrtvu.edu.cn; 2 School of Automation, Guangdong University of Technology, Guangzhou 510006, China

**Keywords:** asymmetric actuator saturation, mecanum-wheeled mobile robot, reinforcement learning, switching event-triggered mechanism

## Abstract

This paper investigates the optimal tracking control problem for a Mecanum-wheeled mobile robot subject to asymmetric actuator saturation. Within the command-filtered backstepping framework, a switching event-triggered control strategy based on reinforcement learning is proposed. First, a compensation system incorporating an anti-saturation term is designed to mitigate both filtering errors and actuator saturation. Then, a novel switching event-triggered mechanism is developed based on the actuator saturation status. It dynamically updates the control signal during unsaturated phases while suspending updates during saturation phases, thereby significantly reducing communication frequency. To achieve optimal control, a reinforcement learning algorithm is constructed by leveraging the function approximation capabilities of neural networks, without relying on prior knowledge of system dynamics. Based on Lyapunov stability theory, it is strictly proven that all signals of the closed-loop system are uniformly ultimately bounded. The simulation results demonstrate that the proposed method effectively reduces data transmission compared with conventional dynamic event-triggered mechanisms, while maintaining satisfactory tracking performance.

## 1. Introduction

A Mecanum-wheeled mobile robot (MWMR), owing to its omnidirectional mobility, can achieve continuous omnidirectional motion without steering the wheels [1]. This capability makes it particularly well-suited for operation in complex environments. Consequently, it has demonstrated significant potential in applications such as autonomous logistics, industrial automation, and hazardous-environment exploration [2,3,4]. Developing efficient and stable adaptive control strategies for MWMR has become a key research focus. However, the inherent strong nonlinearity and coupling in MWMR dynamics pose considerable challenges for controller design [5]. Moreover, practical systems often face energy constraints and actuator saturation, which further complicate the controller design.

Efficient communication and computation are crucial in practical control systems, particularly for resource-constrained mobile robots. Conventional control systems typically employ time-triggered communication. Although this approach is simple to implement, it often results in inefficient resource usage [6,7,8,9]. To address this issue, event-triggered mechanisms have been proposed [10,11,12], which update the control signal only when the system state violates specific conditions, thereby effectively reducing unnecessary signal transmissions. For trajectory tracking of mobile robots, Wang et al. [13] introduced a static event-triggered nonsingular terminal sliding mode controller that achieves finite-time tracking while conserving communication resources. To reduce the complexity of controller design, Chang et al. [14] combined a dynamic event-triggered method with a time-sampling strategy to develop a hybrid dynamic event-triggered mechanism, and applied it to mobile robot formation control. Addressing the leaderless consensus control problem for mobile robots, Xu et al. [15] proposed a novel triggering mechanism based on a cascade structure. This mechanism designs two distinct triggering conditions for angular and position errors, thereby improving system control accuracy. For switched nonlinear systems under communication constraints, Xie et al. [16] developed a switching-mode-based event-triggered control scheme, where each subsystem has an independent triggering condition to conserve network resources under arbitrary switching. Despite these advances, all the aforementioned event-triggered control strategies operate under the implicit assumption that the actuator operates within its linear range. None of them explicitly addresses the case where the actuator enters saturation.

Actuator saturation has also been extensively studied in various practical control systems, including ground vehicle control systems [17], marine surface systems [18], and networked control systems [19], due to its potential to degrade system performance and even cause instability [20,21,22,23,24,25,26]. Most existing research focuses on addressing input constraints to manage actuator saturation [27]. Wang et al. [17] transformed nonlinear saturated inputs into convex combinations of linear terms and designed a tracking controller using robust control techniques. Although this method ensures closed-loop stability under saturation, it may degrade tracking accuracy. To address actuator saturation in marine surface vehicle formation control, Shi et al. [18] proposed a controller based on an auxiliary system but did not consider the asymmetry of constraints. For networked control systems with asymmetric input saturation, Tan et al. [19] used hyperbolic tangent functions to approximate saturation nonlinearity, thereby simplifying controller design. However, this approach may introduce control chattering. Unlike the aforementioned studies, this article introduces a compensation system that directly feeds the saturation error back into the compensation dynamics, effectively mitigating saturation while simultaneously integrating an event-triggered mechanism.

The complex dynamics of MWMR and the high-precision demands of its tasks necessitate not only an efficient anti-saturation scheme but also adaptive and optimal control under model uncertainties [28,29,30,31,32]. For four-wheeled MWMRs with model uncertainties, Chen et al. integrated an adaptive control mechanism with $H_{2}$ optimal control and proposed a nonlinear adaptive optimal control scheme capable of online adaptation to unknown parameter variations, thereby achieving accurate trajectory tracking [33]. Compared to traditional optimal control methods, reinforcement learning demonstrates superior adaptability to complex systems, effectively managing system nonlinearities and uncertainties without requiring complete model knowledge. In recent years, reinforcement learning has been increasingly applied to solve optimal control problems. Juang et al. [34] introduced an interpretable fuzzy reinforcement learning method based on an actor–critic network framework for mobile robot tracking control. Furthermore, for autonomous vehicle systems with safety constraints, Zhang et al. [35] proposed an adaptive safe reinforcement learning algorithm based on a projection operator, which effectively balances safety and optimality. Motivated by the aforementioned literature, we employ an actor–critic RL architecture to directly approximate the gradients of unknown system dynamics and cost functions. This design not only eliminates the need for previous system models, but also enables online learning of optimal strategies under actuator saturation and communication constraints.

To our knowledge, none of these studies have addressed the combined challenges of event-triggered communication, asymmetric actuator saturation, and optimal control within the unified framework of MWMR. Motivated by this gap, this paper focuses on event-triggered optimal control of MWMR with asymmetric actuator saturation within a reinforcement learning framework. The key contributions of this paper are summarized as follows:

- Unlike the event-triggered strategies described in [13,14,15,16], the proposed switching event-triggered mechanism uses a switching signal $\varsigma_{i}$ to smoothly transition between normal and saturated modes, thereby significantly reducing unnecessary communication.
- Within the framework of command-filtered backstepping control, a compensation system with an anti-saturation term is designed to simultaneously address filtering errors and actuator saturation. This approach ensures the control performance of the MWMR system under asymmetric actuator saturation.
- To achieve optimal tracking control without requiring complete knowledge of the MWMR dynamics, an actor–critic reinforcement learning algorithm is developed. This approach reduces reliance on system model knowledge.

## 2. Preliminaries and Problem Formulation

### 2.1. Kinematics and Dynamics of MWMR

To study the motion model of the mobile robot, an inertial reference frame X-O-Y and a body-fixed coordinate system $X_{m}-O_{m}-Y_{m}$ are defined, as illustrated in Figure 1.

The kinematic model of the MWMR is obtained through velocity decomposition and composition as follows:

(1) $$\left[\begin{array}{c} \dot{x}\\ \dot{y}\\ \dot{\theta } \end{array}\right]=\left[\begin{array}{ccc} C_{\theta } & -S_{\theta } & 0\\ S_{\theta } & C_{\theta } & 0\\ 0 & 0 & 1 \end{array}\right]\left[\begin{array}{c} u\\ v\\ \omega \end{array}\right]$$

where *x* and *y* are the MWMR coordinates in the inertial frame, θ represents the orientation angle, and $C_{\theta }=\cos\left(\theta \right)$ and $S_{\theta }=\sin\left(\theta \right)$. *u*, *v*, and ω are the generalized velocities.

By decomposing the velocity of each Mecanum wheel and eliminating intermediate variables, the mapping from the four wheel angular velocities to the generalized velocity of the MWMR is derived as follows:

(2) $$\left[\begin{array}{c} u\\ v\\ \omega \end{array}\right]=\frac{R}{4}\left[\begin{array}{cccc} 1 & 1 & 1 & 1\\ 1 & -1 & 1 & -1\\ \frac{-1}{l_{a}+l_{b}} & \frac{-1}{l_{a}+l_{b}} & \frac{1}{l_{a}+l_{b}} & \frac{1}{l_{a}+l_{b}} \end{array}\right]\left[\begin{array}{c} \omega_{1}\\ \omega_{2}\\ \omega_{3}\\ \omega_{4} \end{array}\right]$$

where $\omega_{i}$(i=1,2,3,4) is the angular velocity of the *i*-th wheel, *R* denotes the wheel radius, and $l_{a}$ and $l_{b}$ stand for the distances from the $O_{m}-Y_{m}$ and $O_{m}-X_{m}$ axes to the wheel center, respectively.

The total kinetic energy consists of the translational and rotational components of the main body, together with the rotational energy of the four wheels, and is computed as

(3) $$K=\frac{1}{2}m\left(u^{2}+v^{2}\right)+\frac{1}{2}I_{a}\omega^{2}+\frac{1}{2}I_{b}\sum_{i=1}^{4}\omega_{i}^{2}$$

where *m*, $I_{a}$, and $I_{b}$ are the MWMR mass, robot inertia, and each wheel’s inertia, respectively.

Assuming the plane of motion corresponds to a zero-potential-energy surface, the total energy can be expressed as follows:

(4) $$\begin{array}{cc} L= & K-S=K\\ = & \frac{1}{2}\left(m\frac{R^{2}}{8}+I_{a}\frac{R^{2}}{16{\left(l_{a}+l_{b}\right)}^{2}}+I_{b}\right)\sum_{i=1}^{4}\omega_{i}^{2}\\ \; & +\left(m\frac{R^{2}}{8}-I_{a}\frac{R^{2}}{16{\left(l_{a}+l_{b}\right)}^{2}}\right)\left(\omega_{1}\omega_{3}+\omega_{2}\omega_{4}\right)\\ \; & -I_{a}\frac{R^{2}}{16{\left(l_{a}+l_{b}\right)}^{2}}\left(\omega_{2}\omega_{3}-\omega_{3}\omega_{4}-\omega_{1}\omega_{2}+\omega_{1}\omega_{4}\right). \end{array}$$

According to the Euler–Lagrange equation, the torque experienced by each Mecanum wheel is given by

(5) $$F_{i}=\frac{d}{dt}\frac{\partial L}{\partial {\dot{\theta }}_{i}}-\frac{\partial L}{\partial \theta_{i}}$$

where ${\dot{\theta }}_{i}=\omega_{i}$. The driving torque $\tau_{si}$ and the static friction torque $\beta_{i}$ satisfy $F_{i}=\tau_{si}-\beta_{i}$, with $\beta_{i}=\mu RN_{i}\;\mathrm{sgn}\left(\omega_{i}\right)$ for i=1,2,3,4.

Considering that the driving torque $\tau_{s}={\left[\tau_{s1},\tau_{s2},\tau_{s3},\tau_{s4}\right]}^{T}$ is subject to asymmetric actuator saturation constraints, the model is given as follows:

(6) $$\tau_{si}=\mathrm{asy}\left(\tau_{i}\right)=\left\{\begin{array}{cc} \tau_{si}^{+}, & \tau_{i}>\tau_{si}^{+}\\ \tau_{i}, & \tau_{si}^{-}\leq \tau_{i}\leq \tau_{si}^{+}\\ \tau_{si}^{-}, & \tau_{i}<\tau_{si}^{-} \end{array}\right.$$

where $\tau_{si}^{+}$ and $\tau_{si}^{-}$ are the saturation bounds with $\tau_{si}^{-}\leq \tau_{si}^{+}$, and $\tau_{i}$ is the intermediate control signal.

From (1)–(5), after algebraic manipulation, we obtain the dynamic equations as follows:

(7) $$\begin{array}{c} \left[\begin{array}{ccc} m+\frac{4I_{b}}{R^{2}} & 0 & 0\\ 0 & m+\frac{4I_{b}}{R^{2}} & 0\\ 0 & 0 & I_{a}+\frac{4{\left(l_{a}+l_{b}\right)}^{2}}{R^{2}} \end{array}\right]\left[\begin{array}{c} \ddot{x}\\ \ddot{y}\\ \ddot{\theta } \end{array}\right]+\left[\begin{array}{c} \frac{4I_{b}}{R^{2}}\dot{x}\dot{\theta }\\ \frac{4I_{b}}{R^{2}}\dot{y}\dot{\theta }\\ 0 \end{array}\right]\\ =\frac{1}{R}{\left[\begin{array}{ccc} C_{\theta }-S_{\theta } & C_{\theta }+S_{\theta } & -(l_{a}+l_{b})\\ C_{\theta }+S_{\theta } & -C_{\theta }+S_{\theta } & -(l_{a}+l_{b})\\ C_{\theta }-S_{\theta } & C_{\theta }+S_{\theta } & l_{a}+l_{b}\\ C_{\theta }+S_{\theta } & -C_{\theta }+S_{\theta } & l_{a}+l_{b} \end{array}\right]}^{T}\left[\begin{array}{c} F_{1}\\ F_{2}\\ F_{3}\\ F_{4} \end{array}\right] \end{array}$$

According to (7), we can obtain the matrix equation

(8) $$M\ddot{P}+C\left(P,\dot{P}\right)+B\beta =B\tau_{s}$$

where $P={\left[x,y,\theta \right]}^{T}$, $\tau_{s}={\left[\tau_{s1},\tau_{s2},\tau_{s3},\tau_{s4}\right]}^{T}$, $C\left(P,\dot{P}\right)={\left[\frac{4I_{b}}{R^{2}}\dot{x}\dot{\theta },\frac{4I_{b}}{R^{2}}\dot{y}\dot{\theta },0\right]}^{T}$, $\beta ={\left[\beta_{1},\beta_{2},\beta_{3},\beta_{4}\right]}^{T}$,

$$B=\frac{1}{R}{\left[\begin{array}{ccc} C_{\theta }-S_{\theta } & C_{\theta }+S_{\theta } & -(l_{a}+l_{b})\\ C_{\theta }+S_{\theta } & -C_{\theta }+S_{\theta } & -(l_{a}+l_{b})\\ C_{\theta }-S_{\theta } & C_{\theta }+S_{\theta } & l_{a}+l_{b}\\ C_{\theta }+S_{\theta } & -C_{\theta }+S_{\theta } & l_{a}+l_{b} \end{array}\right]}^{T},\;M=\left[\begin{array}{ccc} m+\frac{4I_{b}}{R^{2}} & 0 & 0\\ 0 & m+\frac{4I_{b}}{R^{2}} & 0\\ 0 & 0 & I_{a}+\frac{4{\left(l_{a}+l_{b}\right)}^{2}}{R^{2}} \end{array}\right].$$

By defining the velocity vector $V=\dot{P}$, the system (8) is rewritten as

(9) $$\begin{array}{cc} \dot{P}= & V\\ \dot{V}= & M^{-1}B\tau_{s}-M^{-1}C\left(P,\dot{P}\right)-M^{-1}B\beta \end{array}$$

**Assumption A1**  **([**36**]).** *The desired trajectory $P_{r}=\left[x_{r},y_{r},\theta_{r}\right]$ and its first-order derivatives are smooth and bounded.*

### 2.2. Radial Basis Function Neural Network

A radial basis function (RBF) neural network (NN) is used to approximate a nonlinear function $f\left(x\right):R^{v}\to R^{m}$ as [37]

$$f\left(x\right)=W^{*T}\Phi \left(x\right)+\epsilon$$

where $x\in R^{v}$ stands for the input vector, and $\epsilon \in R^{m}$ is the bounded approximation error such that $∥\epsilon ∥\leq \bar{\epsilon }$ with $\bar{\epsilon }>0$. $W^{*}={\left[W_{1}^{*},W_{2}^{*},\ldots ,W_{m}^{*}\right]}^{T}\in R^{n\times m}$ corresponds to the ideal weight matrix

$$W^{*}:=\arg\min_{W\in R^{n\times m}}\left\{\underset{x\in C_{x}}{\sup}∥f\left(x\right)-W^{T}\Phi \left(x\right)∥\right\}$$

where $C_{x}$ represents a sufficiently large operational domain. The basis function vector $\Phi \left(x\right)={\left[\Phi_{1}\left(x\right),\Phi_{2}\left(x\right),\ldots ,\Phi_{n}\left(x\right)\right]}^{T}\in R^{n}$ is defined as:

$$\Phi_{r}\left(x\right)=\exp\left(-\frac{{\left(x-\mu_{r}\right)}^{T}\left(x-\mu_{r}\right)}{\sigma_{r}^{2}}\right)$$

where r=1,2,…,n, $\mu_{r}\in R^{v}$ and $\sigma_{r}$ denote the center and width of the *r*-th basis function, respectively.

### 2.3. Switching Event-Triggered Mechanism

To reduce communication consumption, we design the switching event-triggered mechanism as follows:

(10) $$\begin{array}{cc} \tau_{i} & =\tau_{i}\left(t_{k}\right),\;\forall t\in \left[t_{i}^{k},t_{i}^{k+1}\right)\\ t_{i}^{k+1} & =\inf\left\{t>t_{i}^{k}|\varsigma_{i}|{\tilde{\tau }}_{i}|\;\geq \eta_{i}\left|\tau_{i}\right|+\iota_{i}\right\} \end{array}$$

where $\varsigma_{i}=\frac{1}{2}\mathrm{sgn}\left(\tau_{si}^{+}-\tau_{i}\right)+\frac{1}{2}\mathrm{sgn}\left(\tau_{i}-\tau_{si}^{-}\right)$ represents the switching signal, ${\tilde{\tau }}_{i}=\tau_{i}-\tau_{i}\left(t_{k}\right)$, and ${\dot{\eta }}_{i}=-\varphi_{i}\eta_{i}$ with $0\leq \eta_{i}\left(0\right)\leq 1$. The parameters $\varphi_{i}>0$ and $\iota_{i}>0$ are design parameters. Additionally, $t_{i}^{k}$ ($k\in Z^{+}$) denotes the triggering time.

**Remark 1.** 
*The known switching signal $\varsigma_{i}$ adapts the event-triggered condition to actuator saturation. When the desired control signal lies strictly within the saturation bounds, i.e., $\tau_{si}^{-}<\tau_{i}<\tau_{si}^{+}$, we have $\varsigma_{i}=1$, and the triggering condition reduces to the conventional dynamic event-triggered form $|{\tilde{\tau }}_{i}|\;\geq \eta_{i}\left|\tau_{i}\right|+\iota_{i}$. Hence, events are generated normally.*

*Once $\tau_{i}$ exceeds the upper bound $\tau_{si}^{+}$ or falls below the lower bound $\tau_{si}^{-}$, we obtain $\varsigma_{i}=0$. The left-hand side of the triggering condition becomes identically *0*, while the right-hand side $\eta_{i}\left|\tau_{i}\right|+\iota_{i}$ remains strictly positive. Consequently, the condition can never be satisfied during saturation, and no new event is generated. The actuator input then stays at the saturated value $\tau_{si}^{+}$ or $\tau_{si}^{-}$.*

*This design eliminates futile communication attempts when the actuator cannot respond to updated signals. Without $\varsigma_{i}$, the event condition would keep evaluating $|{\tilde{\tau }}_{i}|\;\geq \eta_{i}\left|\tau_{i}\right|+\iota_{i}$ regardless of saturation, leading to frequent unnecessary triggers. Thus, $\varsigma_{i}$ is essential to suspend event detection during saturation periods.*

*It is worth noting that chattering may occur when the control signal frequently crosses the saturation boundary. The anti-saturation term in the proposed compensation system *(13)* actively mitigates such effects, enhancing robustness in discrete-time or noisy implementations.*

## 3. Main Results

In this section, we design an optimal tracking controller for the MWMR under asymmetric actuator saturation using a reinforcement learning approach within the command-filtered backstepping framework. The overall structure consists of a command filter, a compensation system to address filtering errors and actuator saturation, an RBF NN identifier for unknown dynamics, and an actor–critic NN architecture to approximate the optimal control policy.

Define the tracking errors as

(11) $$\begin{array}{cc} s_{1} & =P-P_{r}\\ s_{2} & =V-\alpha_{f} \end{array}$$

where $\alpha_{f}$ is the filtered output signal generated by the following command filter:

(12) $$\begin{array}{cc} {\dot{\delta }}_{1} & =\gamma_{1}\delta_{2}\\ {\dot{\delta }}_{2} & =-2\gamma_{2}\delta_{2}+\gamma_{1}\left(\alpha_{1}-\delta_{1}\right), \end{array}$$

where $\alpha_{f}=\delta_{1}$, and $\gamma_{1},\gamma_{2}>0$ are design parameters. $\alpha_{1}$ represents the virtual controller.

To compensate for the filtering error and handle actuator saturation, the following compensation system is introduced:

(13) $$\begin{array}{cc} {\dot{\varpi }}_{1} & =-k_{1}\varpi_{1}+\varpi_{2}-\alpha_{1}+\alpha_{f}\\ {\dot{\varpi }}_{2} & =-k_{2}\varpi_{2}-\varpi_{1}+M^{-1}B\left(\tau_{s}-\hat{\tau }\right) \end{array}$$

where $k_{1}$ and $k_{2}$ are positive constants, $\varpi_{1}$ and $\varpi_{2}$ are filter compensating signals, $\tau^{*}$ denotes the optimal control input, and $\hat{\tau }$ is the estimation. The term $M^{-1}B\left(\tau_{s}-\hat{\tau }\right)$ provides an anti-saturation action. When saturation occurs ($\tau_{s}\neq \hat{\tau }$), it actively drives $\varpi_{2}$ to compensate the saturation effect.

The compensated tracking errors are defined as

(14) $$\begin{array}{cc} e_{1} & =s_{1}-\varpi_{1}\\ e_{2} & =s_{2}-\varpi_{2} \end{array}$$

Step 1: From (11)–(14), the time derivative of $e_{1}$ can be calculated as

$$\begin{array}{cc} {\dot{e}}_{1}\; & =V-{\dot{P}}_{r}+k_{1}\varpi_{1}-\varpi_{2}+\alpha_{1}-\alpha_{f}\\ \; & =e_{2}+\alpha_{1}-{\dot{P}}_{r}+k_{1}\varpi_{1} \end{array}$$

Consider the following Lyapunov function candidate:

(15) $$\begin{array}{c} V_{1}=\frac{1}{2}e_{1}^{T}e_{1} \end{array}$$

Its derivative is

(16) $${\dot{V}}_{1}=e_{1}^{T}\left(e_{2}+\alpha_{1}-{\dot{P}}_{r}+k_{1}\varpi_{1}\right)$$

Applying Young’s inequality, it yields

(17) $$e_{1}^{T}e_{2}\leq \frac{1}{2}e_{1}^{T}e_{1}+\frac{1}{2}e_{2}^{T}e_{2}$$

The virtual controller is designed as follows:

(18) $$\alpha_{1}=-k_{1}s_{1}-\frac{1}{2}e_{1}+{\dot{P}}_{r}$$

Substituting (17) and (18) into (16), we obtain

(19) $${\dot{V}}_{1}\leq -k_{1}e_{1}^{T}e_{1}+\frac{1}{2}e_{2}^{T}e_{2}$$

Step 2: From (11)–(14), the time derivative of $e_{2}$ can be computed as

(20) $$\begin{array}{cc} {\dot{e}}_{2} & ={\dot{s}}_{2}-{\dot{\varpi }}_{2}\\ \; & =\left(M^{-1}B\tau_{s}+\Gamma_{0}\right)-\left(-k_{2}\varpi_{2}-\varpi_{1}+M^{-1}B\left(\tau_{s}-\hat{\tau }\right)\right)\\ \; & =M^{-1}B\hat{\tau }+\Gamma +k_{2}\varpi_{2} \end{array}$$

where $\Gamma_{0}=-M^{-1}C\left(P,\dot{P}\right)-M^{-1}B\beta -{\dot{\alpha }}_{f}$ and $\Gamma =\Gamma_{0}+\varpi_{1}$.

Define the following cost function as

(21) $$\begin{array}{cc} J_{2}^{*}= & \min_{\tau \in \Omega_{\tau }}\int_{t}^{\infty }e^{-\gamma (\xi -t)}\left(e_{2}^{T}Qe_{2}+\tau^{T}R\tau \right)d\xi \\ = & \int_{t}^{\infty }e^{-\gamma (\xi -t)}\left(e_{2}^{T}e_{2}+\tau^{*T}{\left(M^{-1}B\right)}^{T}\left(M^{-1}B\right)\tau^{*}\right)d\xi \end{array}$$

where Q and R are symmetric positive definite matrices, γ>0 is a discount factor, and $\Omega_{\tau }$ denotes the set of admissible controls.

**Remark 2.** 
*Let $Q=I_{3\times 3}$ and $R={\left(M^{-1}B\right)}^{T}\left(M^{-1}B\right)$. Since $M^{-1}B\in R^{3\times 4}$ is not a square matrix, its regular inverse does not exist. To overcome this issue, we employ the Moore–Penrose pseudo-inverse denoted by ${\left(M^{-1}B\right)}^{†}$, which satisfies $\left(M^{-1}B\right){\left(M^{-1}B\right)}^{†}=I_{3\times 3}$. As discussed in [37], using the pseudo-inverse yields a unique and bounded control signal. Therefore, we employ the pseudo-inverse in the following derivations. Note that the explicit expression for the pseudo-inverse is ${\left(M^{-1}B\right)}^{†}={\left(M^{-1}B\right)}^{T}{\left(\left(M^{-1}B\right){\left(M^{-1}B\right)}^{T}\right)}^{-1}$, and for the matrix B we have $B^{†}=B^{T}{\left(BB^{T}\right)}^{-1}$ because B is row-full rank. Moreover, ${\left(M^{-1}B\right)}^{†}=B^{†}M$ holds since M is invertible.*

According to reference [34], the Hamiltonian function is defined as

(22) $$\begin{array}{cc} H_{2}^{*}\left(e_{2},\tau^{*},\frac{\partial J_{2}^{*}}{\partial e_{2}}\right)= & e_{2}^{T}e_{2}+\tau^{*T}{\left(M^{-1}B\right)}^{T}\left(M^{-1}B\right)\tau^{*}-\gamma J_{2}\\ & \;+\frac{\partial J_{2}^{T}}{\partial e_{2}}\left(M^{-1}B\tau^{*}+\Gamma +k_{2}\varpi_{2}\right) \end{array}$$

Since $\partial H_{2}^{*}/\partial \tau^{*}=0$, the optimal control input is deduced as follows:

(23) $$\tau^{*}=-\frac{1}{2}{\left(M^{-1}B\right)}^{†}\frac{\partial J_{2}^{*}}{\partial e_{2}}$$

To achieve the optimal control, the gradient of the cost function $\frac{\partial J_{2}^{*}}{\partial e_{2}}$ is segmented as follows:

(24) $$\frac{\partial J_{2}^{*}}{\partial e_{2}}=\frac{5}{2}e_{2}+2k_{2}s_{2}+2\Gamma +J_{2}^{0}$$

where $J_{2}^{0}=\frac{\partial J_{2}^{*}}{\partial e_{2}}-3e_{2}-2k_{2}s_{2}-2\Gamma$.

From (23) and (24), using ${\left(M^{-1}B\right)}^{†}=B^{†}M$, the optimal control input can be written as

(25) $$\tau^{*}=-B^{†}M\left(\frac{5}{4}e_{2}+k_{2}s_{2}+\Gamma +\frac{1}{2}J_{2}^{0}\right)$$

The nonlinear function Γ and $J_{2}^{0}$ are approximated by RBF NNs:

(26) $$\begin{array}{cc} \Gamma = & W^{*T}\Phi \left(\chi \right)+\varepsilon \\ J_{2}^{0}= & W_{p}^{*T}\Phi_{p}\left(\chi \right)+\varepsilon_{p} \end{array}$$

where $\chi ={\left[P^{T},V^{T}\right]}^{T}\in R^{6}$ is the input vector. For ease of notation, write Φ(χ) as Φ and $\Phi_{p}\left(\chi \right)$ as $\Phi_{p}$. ε ad $\varepsilon_{p}$ denote the bounded approximation errors satisfying $∥\varepsilon ∥\leq \bar{\varepsilon }$ and $∥\varepsilon_{p}∥\leq {\bar{\varepsilon }}_{p}$ with $\bar{\varepsilon },{\bar{\varepsilon }}_{p}>0$.

Substituting (26) into (24) and (25), we have

(27) $$\frac{\partial J_{2}^{*}}{\partial e_{2}}=\frac{5}{2}e_{2}+2k_{2}s_{2}+2W^{*T}\Phi +W_{p}^{*T}\Phi_{p}+2\varepsilon +\varepsilon_{p}$$

(28) $$\tau^{*}=-B^{†}M\left(\frac{5}{4}e_{2}+k_{2}s_{2}+W^{*T}\Phi +\varepsilon +\frac{1}{2}W_{p}^{*T}\Phi_{p}+\frac{1}{2}\varepsilon_{p}\right)$$

By using a reinforcement learning algorithm, we have

(29) $$\frac{\partial {\hat{J}}_{2}}{\partial e_{2}}=\frac{5}{2}e_{2}+2k_{2}s_{2}+2{\hat{W}}^{T}\Phi +{\hat{W}}_{c}^{T}\Phi_{p}$$

(30) $$\hat{\tau }=-B^{†}M\left(\frac{5}{4}e_{2}+k_{2}s_{2}+{\hat{W}}^{T}\Phi +\frac{1}{2}{\hat{W}}_{a}^{T}\Phi_{p}\right)$$

where $\hat{W}$, ${\hat{W}}_{c}$, and ${\hat{W}}_{a}$ are estimates of $W^{*}$, $W_{p}^{*}$ (for the critic), and $W_{p}^{*}$ (for the actor), respectively.

The adaptive updating laws are designed as follows:

(31) $$\dot{\hat{W}}=\Phi e_{2}^{T}-h\hat{W}$$

(32) $${\dot{\hat{W}}}_{c}=-\kappa_{c}\Phi_{p}\Phi_{p}^{T}{\hat{W}}_{c}$$

(33) $${\dot{\hat{W}}}_{a}=-\kappa_{a}\Phi_{p}\Phi_{p}^{T}\left({\hat{W}}_{a}-{\hat{W}}_{c}\right)$$

where h>0, $\kappa_{a}>\frac{1}{2}$ and $\kappa_{c}>2\kappa_{a}$ are design parameters.

Choose the following Lyapunov function:

(34) $$V_{2}=\frac{e_{2}^{T}e_{2}}{2}+\frac{\mathrm{tr}({\tilde{W}}^{T}\tilde{W})}{2}+\frac{\mathrm{tr}({\tilde{W}}_{c}^{T}{\tilde{W}}_{c})}{2}+\frac{\mathrm{tr}({\tilde{W}}_{a}^{T}{\tilde{W}}_{a})}{2}$$

where $\tilde{W}=W^{*}-\hat{W}$, ${\tilde{W}}_{c}=W_{c}^{*}-{\hat{W}}_{c}$, ${\tilde{W}}_{a}=W_{a}^{*}-{\hat{W}}_{a}$.

According to (20) and (30)–(33), the time derivative of $V_{2}$ can be calculated as follows:

(35) $$\begin{array}{cc} {\dot{V}}_{2}\leq & -\left(k_{2}+\frac{1}{2}\right)e_{2}^{T}e_{2}+\kappa_{c}\mathrm{tr}\left({\tilde{W}}_{c}^{T}\Phi_{p}\Phi_{p}^{T}{\hat{W}}_{c}\right)+h\mathrm{tr}\left({\tilde{W}}^{T}\hat{W}\right)+\kappa_{a}\mathrm{tr}\left({\tilde{W}}_{a}^{T}\Phi_{p}\Phi_{p}^{T}{\hat{W}}_{a}\right)\\ \; & -\kappa_{a}\mathrm{tr}\left({\tilde{W}}_{a}^{T}\Phi_{p}\Phi_{p}^{T}{\hat{W}}_{c}\right)+\frac{1}{4}{∥{\hat{W}}_{a}^{T}\Phi_{p}∥}^{2}+\frac{1}{2}{\bar{\varepsilon }}^{2} \end{array}$$

**Remark 3.** 
*In practice, the proposed controller involves several parameters that must be selected carefully. Typically, increasing $k_{1}$ and $k_{2}$ accelerates the convergence of tracking errors, but excessively large values may cause saturation. For the command filters, $\gamma_{1}$ determines the response speed, while $\gamma_{2}$ affects damping. These parameters are usually set within the range [0.5, 5] in our simulations. The event-triggered parameters $\varphi_{i}$ and $\iota_{i}$ balance communication reduction and tracking performance: a larger $\iota_{i}$ may reduce the number of triggers but can also degrade accuracy. A practical tuning procedure is to first adjust $k_{1}$ and $k_{2}$ to achieve acceptable tracking, then tune $\gamma_{1}$ and $\gamma_{2}$ for smoothness, and finally modify $\varphi_{i}$ and $\iota_{i}$ to meet communication requirements. The parameters used in our simulations are summarized at the beginning of Section 5 as a reference.*

## 4. Stability Analysis

**Theorem 1.** 
*Under Assumption 1, for the MWMR system (9) subject to actuator saturation, the proposed control scheme consists of the switching event-triggered mechanism (10), the compensation system (13), the virtual controller (18), the optimal control input (30), and the adaptive update laws (31)–(33). By selecting appropriate design parameters, all error signals ($e_{1}$, $e_{2}$, $\tilde{W}$, ${\tilde{W}}_{c}$, and ${\tilde{W}}_{a}$) are bounded. Moreover, the Zeno phenomenon is excluded.*

**Proof.**  Consider the following Lyapunov function candidate:

(36) $$V=V_{1}+V_{2}$$

According to (19), (35) and (36), we have

(37) $$\begin{array}{cc} \dot{V}\leq & -k_{1}e_{1}^{T}e_{1}-k_{2}e_{2}^{T}e_{2}+\kappa_{c}\mathrm{tr}({\tilde{W}}_{c}^{T}\Phi_{p}\Phi_{p}^{T}{\hat{W}}_{c})-h\mathrm{tr}\left({\tilde{W}}^{T}\hat{W}\right)+\kappa_{a}\mathrm{tr}({\tilde{W}}_{a}^{T}\Phi_{p}\Phi_{p}^{T}{\hat{W}}_{a})\\ \; & -\kappa_{a}\mathrm{tr}\left({\tilde{W}}_{a}^{T}\Phi_{p}\Phi_{p}^{T}{\hat{W}}_{c}\right)+\frac{1}{4}{∥{\hat{W}}_{a}^{T}\Phi_{p}∥}^{2}+\frac{1}{2}{\bar{\varepsilon }}^{2} \end{array}$$

Using Young’s inequality yields

(38) $$\begin{array}{cc} \mathrm{tr}({\tilde{W}}^{T}\hat{W})\leq - & \frac{\mathrm{tr}({\tilde{W}}^{T}\tilde{W})}{2}+\frac{\mathrm{tr}(W^{*T}W^{*})}{2}\\ -\kappa_{a}\mathrm{tr}\left({\tilde{W}}_{a}^{T}\Phi_{p}\Phi_{p}^{T}{\hat{W}}_{c}\right)\leq & \frac{\kappa_{a}}{4}\mathrm{tr}\left({\tilde{W}}_{a}^{T}\Phi_{p}\Phi_{p}^{T}{\tilde{W}}_{a}\right)+\kappa_{a}\mathrm{tr}\left({\hat{W}}_{c}^{T}\Phi_{p}\Phi_{p}^{T}{\hat{W}}_{c}\right) \end{array}$$

Moreover, utilizing ${\tilde{W}}_{a}=W_{p}^{*}-{\hat{W}}_{a}$ and ${\tilde{W}}_{c}=W_{p}^{*}-{\hat{W}}_{c}$ gives

(39) $${\tilde{W}}_{a}^{T}\Phi_{p}\Phi_{p}^{T}{\hat{W}}_{a}=-\frac{{\tilde{W}}_{a}^{T}\Phi_{p}\Phi_{p}^{T}{\tilde{W}}_{a}}{2}-\frac{{\hat{W}}_{a}^{T}\Phi_{p}\Phi_{p}^{T}{\hat{W}}_{a}}{2}+\frac{W_{a}^{*T}\Phi_{p}\Phi_{p}^{T}W_{a}^{*}}{2}$$

(40) $${\tilde{W}}_{c}^{T}\Phi_{p}\Phi_{p}^{T}{\hat{W}}_{c}=-\frac{{\tilde{W}}_{c}^{T}\Phi_{p}\Phi_{p}^{T}{\tilde{W}}_{c}}{2}-\frac{{\hat{W}}_{c}^{T}\Phi_{p}\Phi_{p}^{T}{\hat{W}}_{c}}{2}+\frac{W_{c}^{*T}\Phi_{p}\Phi_{p}^{T}W_{c}^{*}}{2}$$

Substituting (38)–(40) into (37), after collecting terms we obtain

(41) $$\begin{array}{cc} \dot{V}\leq \; & -k_{1}e_{1}^{T}e_{1}-k_{2}e_{2}^{T}e_{2}-\frac{h}{2}\mathrm{tr}\left({\tilde{W}}^{T}\tilde{W}\right)-\frac{\kappa_{a}}{4}\mathrm{tr}\left({\tilde{W}}_{a}^{T}\Phi_{p}\Phi_{p}^{T}{\tilde{W}}_{a}\right)-\frac{\kappa_{c}}{2}\mathrm{tr}\left({\tilde{W}}_{c}^{T}\Phi_{p}\Phi_{p}^{T}{\tilde{W}}_{c}\right)\\ \; & -\frac{\kappa_{c}-2\kappa_{a}}{2}\mathrm{tr}\left({\hat{W}}_{c}^{T}\Phi_{p}\Phi_{p}^{T}{\hat{W}}_{c}\right)-\frac{2\kappa_{a}-1}{4}\mathrm{tr}\left({\hat{W}}_{a}^{T}\Phi_{p}\Phi_{p}^{T}{\hat{W}}_{a}\right)+\frac{h}{2}\mathrm{tr}\left(W^{*T}W^{*}\right)\\ & +\frac{\kappa_{a}}{2}\mathrm{tr}\left(W_{a}^{*T}\Phi_{p}\Phi_{p}^{T}W_{a}^{*}\right)+\frac{\kappa_{c}}{2}\mathrm{tr}\left(W_{c}^{*T}\Phi_{p}\Phi_{p}^{T}W_{c}^{*}\right)+\frac{1}{2}{\bar{\varepsilon }}^{2} \end{array}$$

Then, (41) becomes

(42) $$\begin{array}{c} \dot{V}\leq -CV+M \end{array}$$

where $C=\min\{2k_{1},2k_{2},h,\kappa_{c},\frac{\kappa_{a}}{2}\}$, $M=\frac{1}{2}{\bar{\varepsilon }}^{2}+\frac{\kappa_{c}}{2}\mathrm{tr}\left(W_{c}^{*T}\Phi_{p}\Phi_{p}^{T}W_{c}^{*}\right)+\frac{\kappa_{a}}{2}\mathrm{tr}\left(W_{a}^{*T}\Phi_{p}\Phi_{p}^{T}W_{a}^{*}\right)$$+\frac{h}{2}\mathrm{tr}\left(W^{*T}W^{*}\right)$.

Integrating both sides of (42) yields

(43) $$\begin{array}{c} V\leq \left(V\left(0\right)-\frac{M}{C}\right)e^{-Ct}+\frac{M}{C}, \end{array}$$

which implies that all signals are bounded.

Next, we exclude Zeno behavior and consider the following two cases.

Case I (With actuator saturation): This case occurs when $\tau_{i}\leq \tau_{si}^{-}$ or $\tau_{i}\geq \tau_{si}^{+}$.

The input to the actuator is a saturation value, $\varsigma_{i}=0$, and the triggering condition $\varsigma_{i}|{\tilde{\tau }}_{i}|\;\geq \eta_{i}\left|\tau_{i}\right|+\iota_{i}$ simplifies to $0\geq \eta_{i}\left|\tau_{i}\right|+\iota_{i}$, which is never satisfied. Therefore, no event is generated.

Case II (Without actuator saturation): This case occurs when $\tau_{si}^{-}<\tau_{i}<\tau_{si}^{+}$.

For the control signal $\tau_{i}$, assuming that Zeno behavior exists, there must be a finite time instant $T_{i}>0$ such that $\lim_{k\to \infty }t_{i}^{k}=T_{i}$.

For any $k\in Z^{+}$, during the interval $\left[t_{i}^{k},t_{i}^{k+1}\right)$, $|{\tilde{\tau }}_{i}|$ initially equals zero and may later reach zero multiple times. Let ${\bar{t}}_{i}^{\kappa }$ be the last time instant where $|{\tilde{\tau }}_{i}|=0$, with $t_{i}^{k}\leq {\bar{t}}_{i}^{\kappa }\leq t_{i}^{k+1}$. Consequently, we can compute the derivative of $|{\tilde{\tau }}_{i}|$ on $\left[{\bar{t}}_{i}^{\kappa },t_{i}^{k+1}\right)$ as follows:

(44) $$\begin{array}{c} \frac{d}{dt}|{\tilde{\tau }}_{i}|={\dot{\tilde{\tau }}}_{i}\mathrm{sgn}\left({\tilde{\tau }}_{i}\right)\leq \left|{\dot{\tau }}_{i}\right| \end{array}$$

From the definition of $\tau_{i}$, one can find a positive constant $\rho_{i}$ such that $\frac{d}{dt}|{\tilde{\tau }}_{i}\left|\leq \right|{\dot{\tau }}_{i}|\leq \rho_{i}$. According to (10), when $t\to t_{i}^{k+1}$, a sufficient condition for an event to occur is $\lim_{t\to t_{i}^{k+1}}\left|{\tilde{\tau }}_{i}\right|\geq \iota_{i}$. Since ${\tilde{\tau }}_{i}\left({\bar{t}}_{i}^{\kappa }\right)=0$, from (44), the trigger interval satisfies

(45) $$\begin{array}{c} t_{i}^{k+1}-t_{i}^{k}\geq t_{i}^{k+1}-{\bar{t}}_{i}^{\kappa }\geq \frac{\iota_{i}}{\rho_{i}}. \end{array}$$

This precludes the occurrence of infinitely many events in any finite interval $\left[0,T_{i}\right]$. Hence, Zeno behavior is precluded. □

## 5. Simulation Result

In this section, three simulation scenarios are designed to comprehensively evaluate the proposed reinforcement learning-based switching event-triggered tracking controller. The proposed method is compared with two representative algorithms from the literature [13,19] under three distinct reference trajectories to verify its generality and effectiveness.

The MWMR parameters are set as follows: m=5 kg, $l_{a}=0.3$ m, $l_{b}=0.3$ m, $I_{a}=5$ kg·m^2^, $I_{b}=0.1$ kg·m^2^, R=0.05 m, $N_{i}=0.25$ mg, and g=9.8 m/s^2^. The sampling period is $T_{s}=0.001$ s. For the proposed method, the event-triggering parameters are set to $\varphi_{i}=0.1$ and $\iota_{i}=0.02$ for i=1,…,4, and the learning rates are h=1, $\kappa_{a}=1.2$, $\kappa_{c}=3$. The command filter parameters are $\gamma_{1}=1$ and $\gamma_{2}=3$ for all cases.

Case 1: The initial states are $P\left(0\right)={\left[0.5,\;-0.3,\;0\right]}^{T}$ m and $V\left(0\right)={\left[0,\;0,\;0\right]}^{T}$ m/s. The asymmetric saturation limits are $\tau_{si}^{+}=0.8$ Nm and $\tau_{si}^{-}=-0.5$ Nm. The reference trajectory is given by $P_{r}={\left[10\sin\left(0.06t\right),\;5\sin\left(0.12t\right),\;\arctan\left({\dot{y}}_{r},{\dot{x}}_{r}\right)\right]}^{T}$. The controller gains for the proposed method are $k_{1}=8$ and $k_{2}=10$.

The simulation results for Case 1 are presented in Figure 2, Figure 3, Figure 4 and Figure 5. Figure 2 compares the tracking trajectories obtained using our proposed method with those from the methods described in [12,19]. It can be observed that all three methods successfully achieve trajectory tracking. Figure 3 depicts the time evolution of the tracking errors $e_{x}$, $e_{y}$, and $e_{\theta }$, demonstrating that our proposed controller achieves faster convergence and smaller tracking errors. Figure 4 plots the trajectories of each motor torque. Figure 5 presents the convergence of the actor–critic network weight norms for this paper. The norms for all output dimensions become stable over time, demonstrating that the neural networks successfully adapt online to the unknown robot dynamics.

Table 1 and Table 2 present the RMSE of tracking errors and the number of triggering events for all three methods, respectively. The proposed method achieves the lowest RMSE among the three methods while maintaining the fewest triggering events, demonstrating that the switching event-triggered mechanism effectively reduces communication load without compromising control performance.

Case 2: The initial states are $P\left(0\right)={\left[0,\;0.3,\;0.1\right]}^{T}$ m and $V\left(0\right)={\left[0,\;0,\;0\right]}^{T}$ m/s. The asymmetric saturation limits are $\tau_{si}^{+}=0.6$ Nm and $\tau_{si}^{-}=-0.4$ Nm. The reference trajectory is a spiral given by $P_{r}={\left[r\cos\left(0.1t\right),\;r\sin\left(0.1t\right),\;\arctan\left({\dot{y}}_{r},{\dot{x}}_{r}\right)\right]}^{T},$ with r=0.5+0.05t. The controller gains for the proposed method are $k_{1}=5$ and $k_{2}=20$.

Figure 6 and Figure 7 and Table 3 and Table 4 present the results for Case 2. Figure 6 compares the tracking trajectories, and Figure 7 shows the tracking errors. The proposed method again achieves the smallest tracking errors and the lowest trigger counts among the three methods, as quantified in Table 3 and Table 4.

Case 3: The initial states are $P\left(0\right)={\left[-0.5,\;-0.2,\;0.1\right]}^{T}$ m and $V\left(0\right)={\left[0,\;0,\;0\right]}^{T}$ m/s. The asymmetric saturation limits are $\tau_{si}^{+}=0.3$ Nm and $\tau_{si}^{-}=-0.5$ Nm. The reference trajectory is given by $P_{r}={\left[0.3t+1,\;4\sin\left(0.08t\right)+2\sin\left(0.12t\right)+1.5\cos\left(0.06t\right)+0.8\sin\left(0.15t\right),\;\arctan\left({\dot{y}}_{r},{\dot{x}}_{r}\right)\right]}^{T}$. The controller gains are $k_{1}=3$ and $k_{2}=10$.

Figure 8 and Figure 9 and Table 5 and Table 6 present the results for Case 3. The proposed method consistently achieves the best tracking accuracy and the lowest communication load among the three methods.

The simulation results from the three cases consistently demonstrate the superiority of the proposed method. Across all three trajectories with different geometric characteristics and saturation constraints, the proposed switching event-triggered reinforcement learning controller achieves the smallest tracking errors and the lowest number of triggering events compared to the two representative algorithms from the literature. The proposed method thus provides a robust and efficient solution for MWMR trajectory tracking under asymmetric actuator saturation.

## 6. Conclusions

This paper has investigated the optimal trajectory tracking control problem for an MWMR subject to asymmetric actuator saturation. Within the command-filtered backstepping framework, a switching event-triggered control scheme based on reinforcement learning has been proposed. A compensation system with an anti-saturation term is designed to simultaneously compensate filtering errors and actuator saturation, ensuring closed-loop stability without requiring prior knowledge of the saturation nonlinearity. Unlike conventional event-triggered strategies, the designed switching mechanism suspends control signal updates when the actuator enters saturation and resumes them only when the saturation is relieved, thereby avoiding unnecessary communication during saturation periods. Furthermore, the designed reinforcement learning algorithm enables online learning of the optimal control policy. The simulation results show that the proposed method achieves satisfactory tracking performance while significantly reducing communication resource waste.

## Figures and Tables

**Figure 1:**
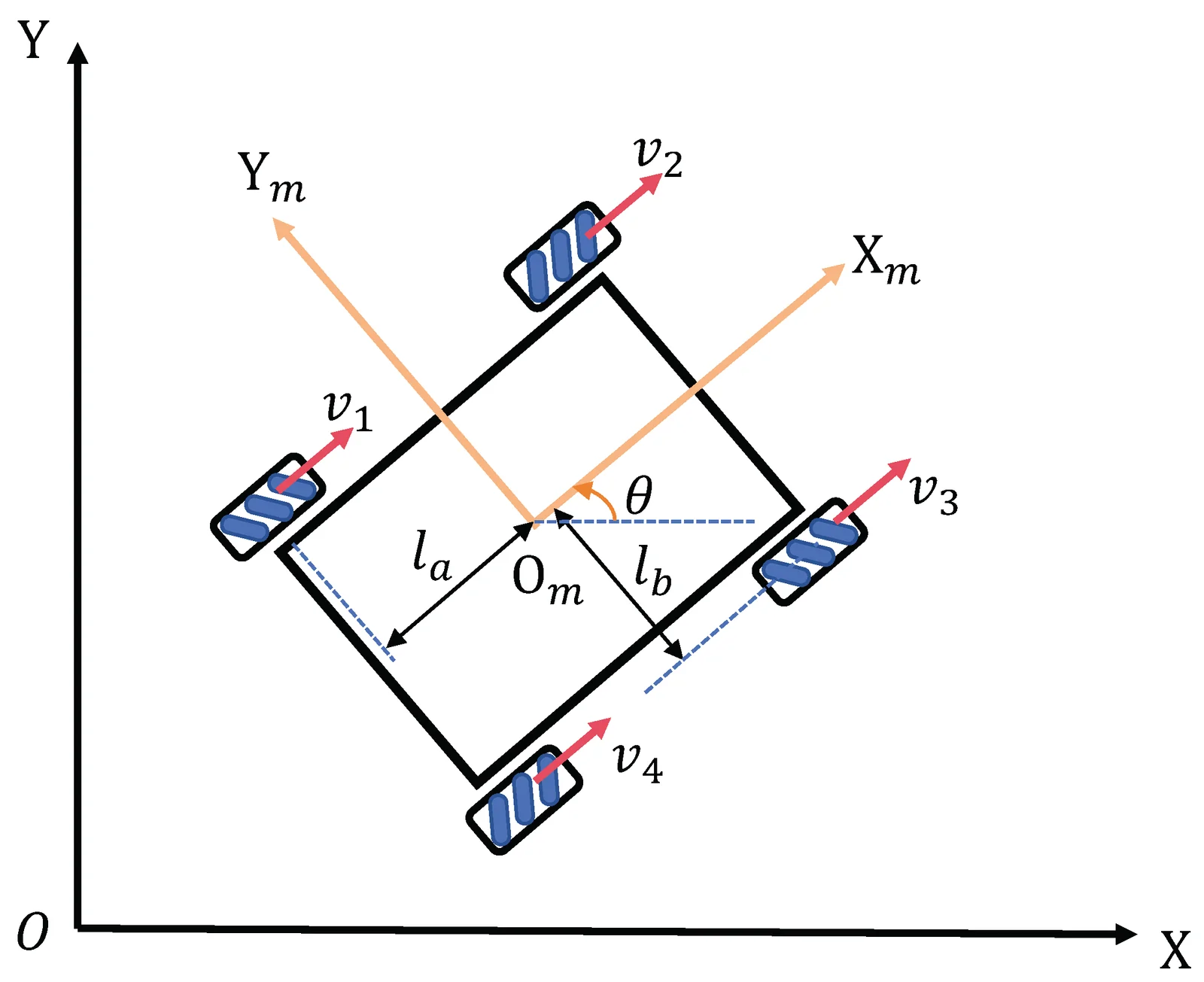
Kinematic model of the MWMR.

**Figure 2:**
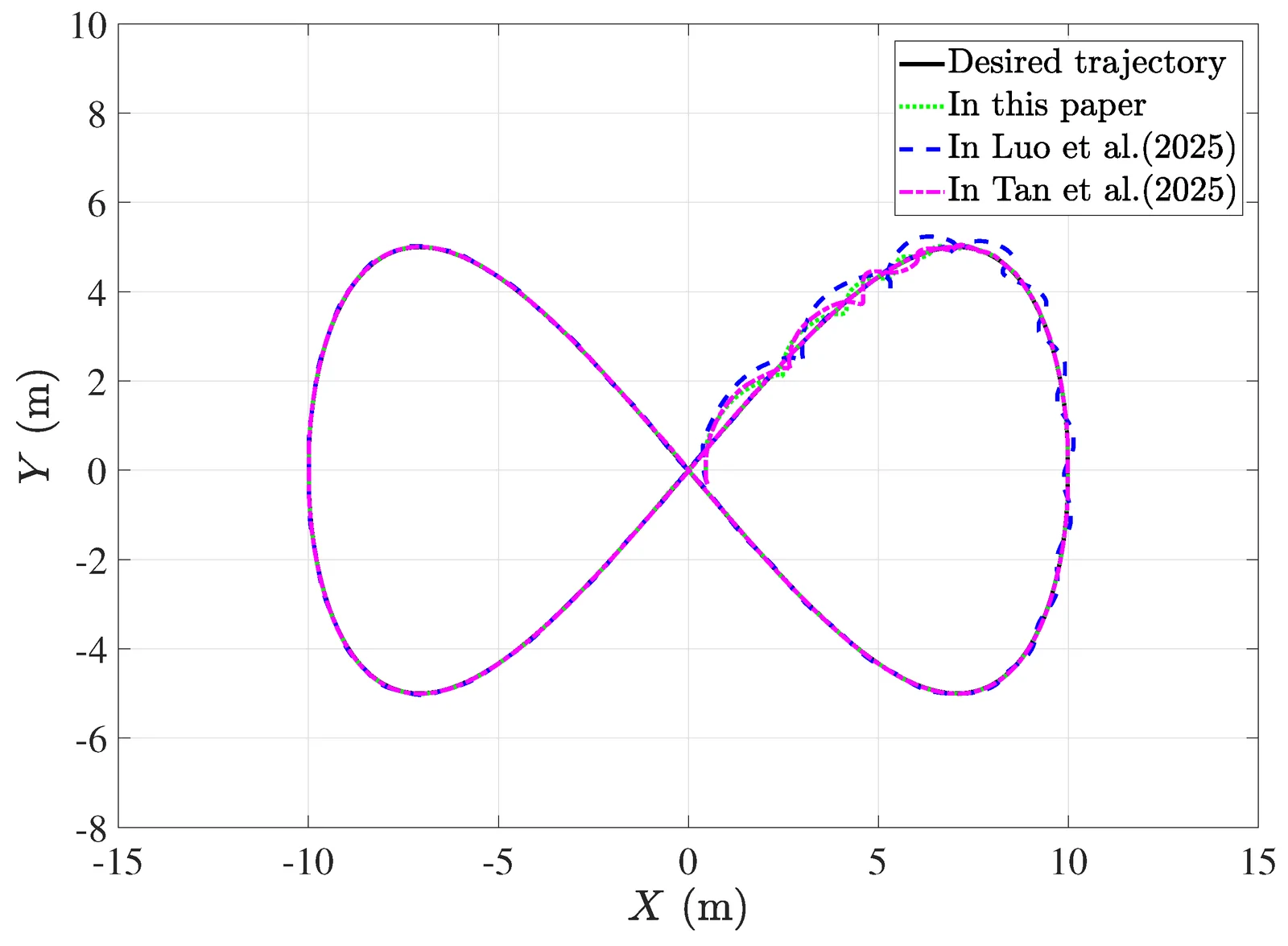
The tracking trajectories of the three methods in this paper, [12], and [19] for Case 1.

**Figure 3:**
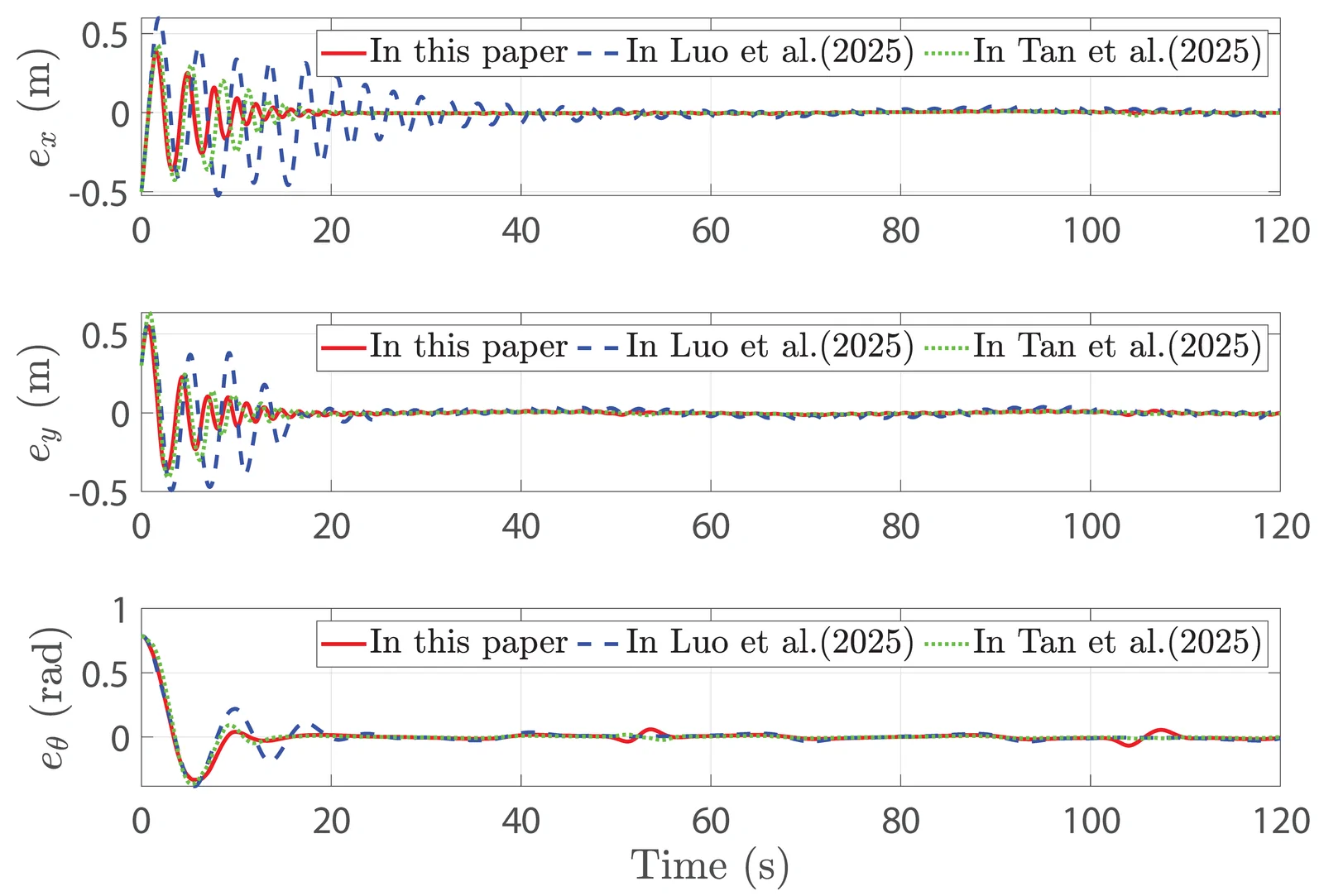
The trajectories of tracking errors in this paper, [12], and [19] for Case 1.

**Figure 4:**
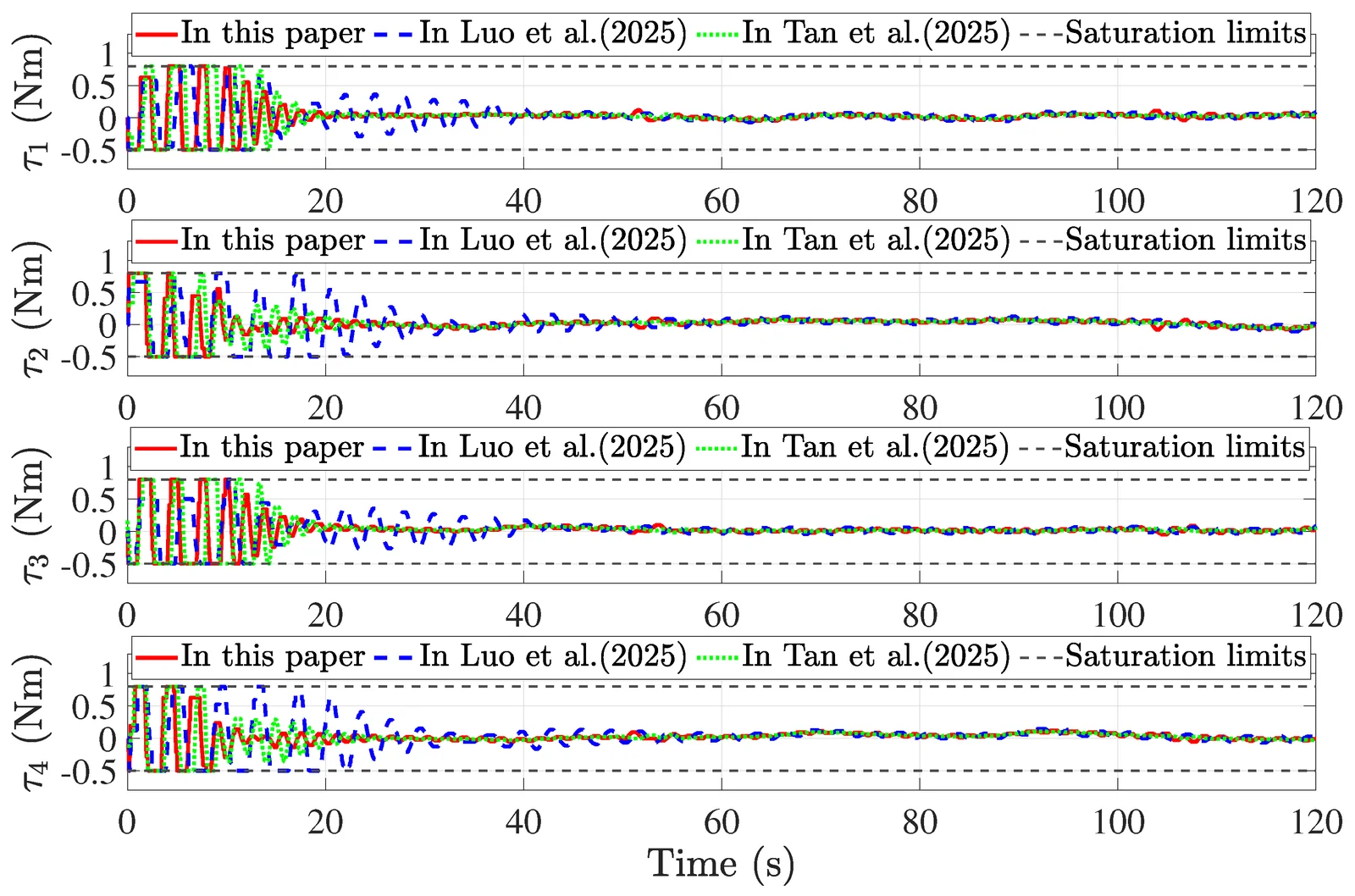
The trajectories of each motor torque in this paper, [12], and [19] for Case 1.

**Figure 5:**
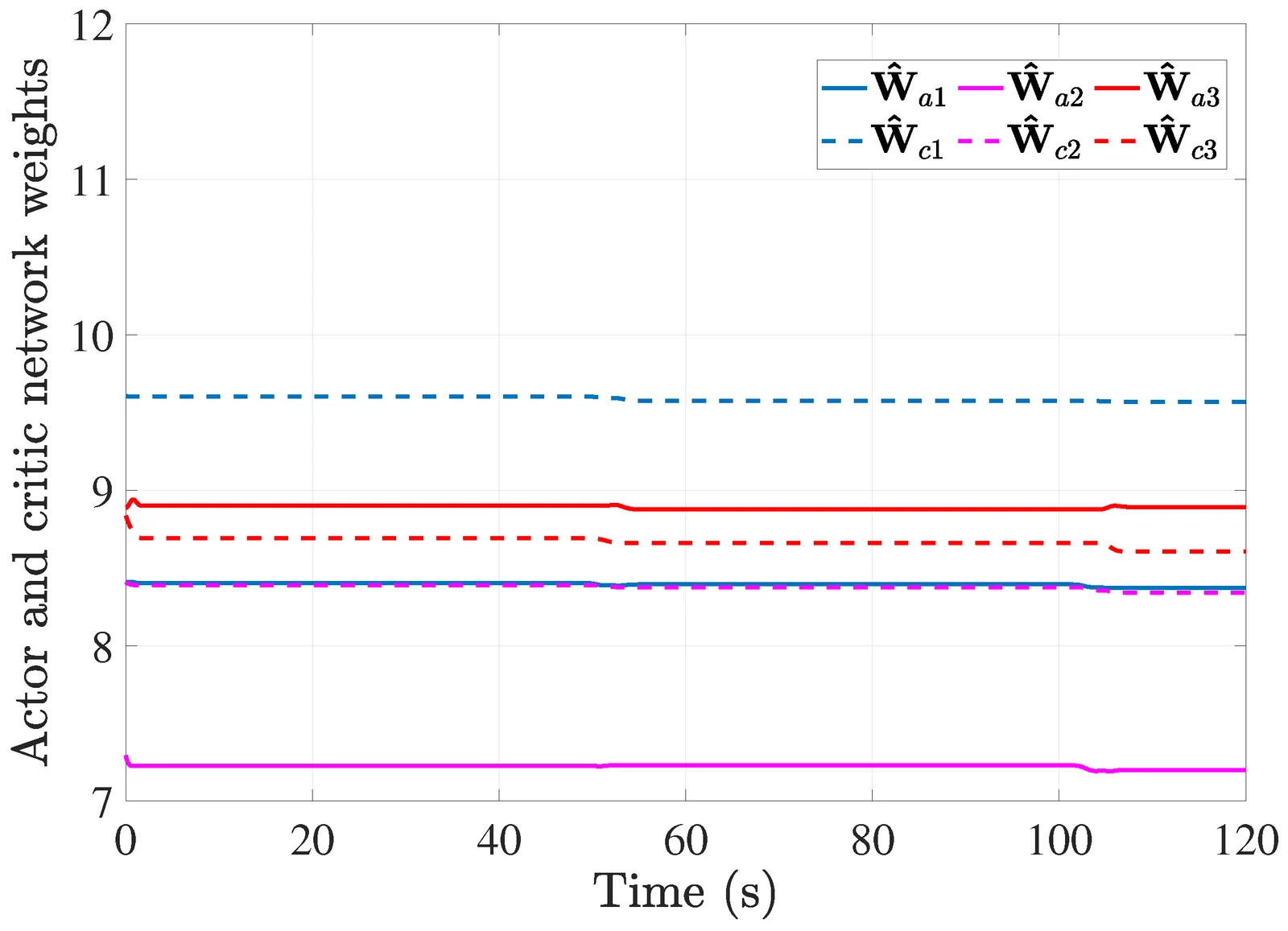
The trajectories of actor–critic network weight norms for Case 1.

**Figure 6:**
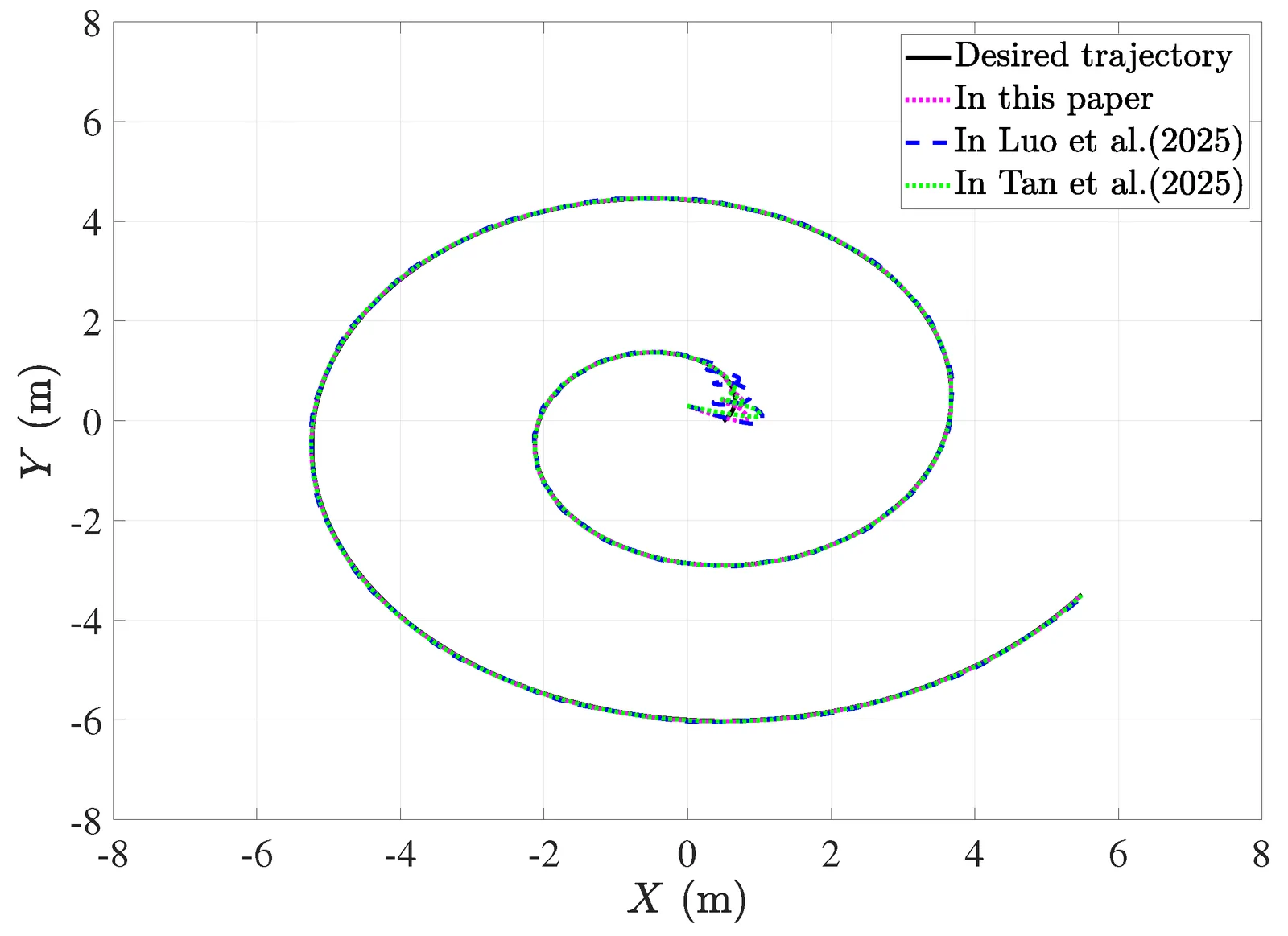
The tracking trajectories of the three methods in this paper, [12], and [19] for Case 2.

**Figure 7:**
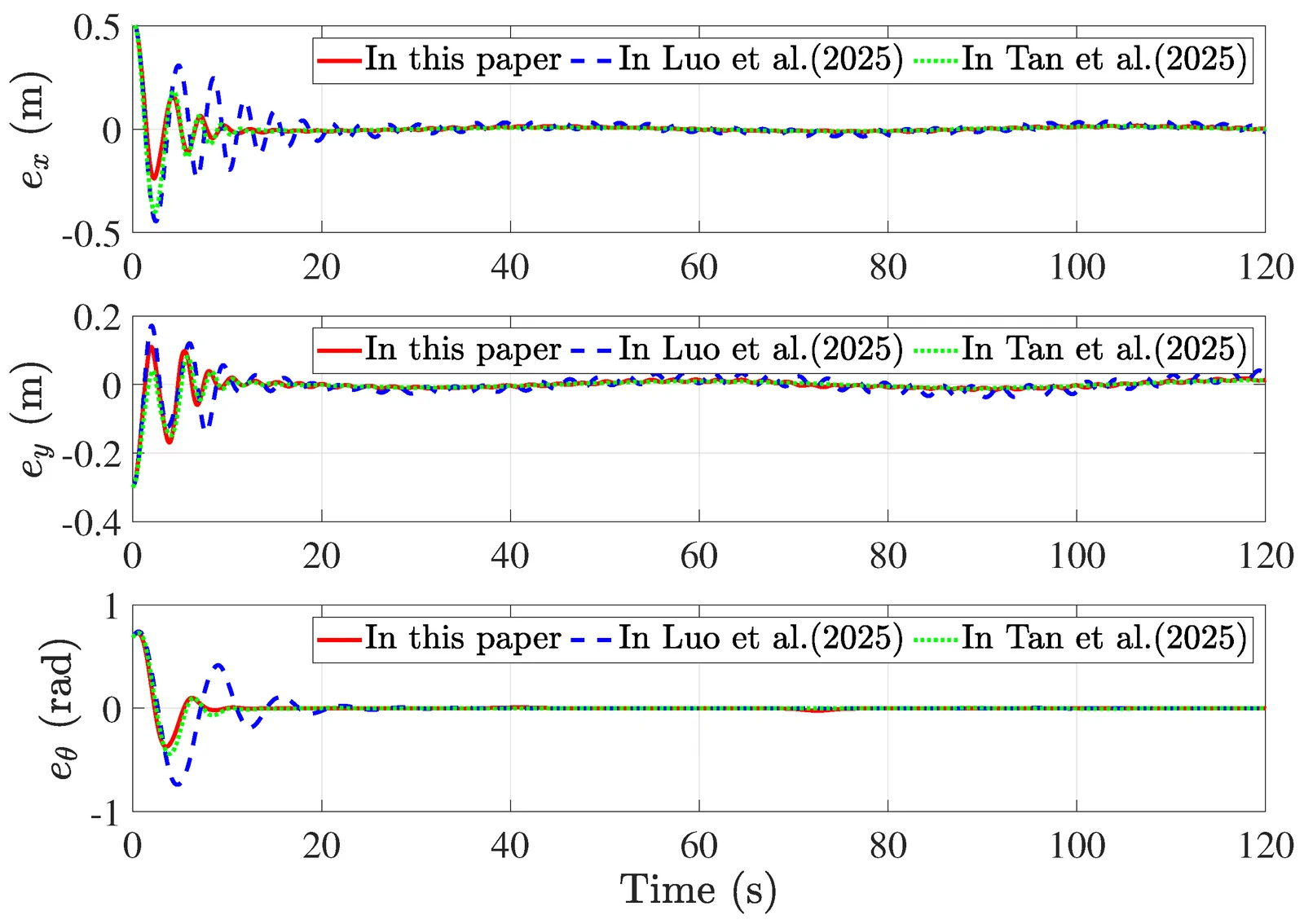
The trajectories of tracking errors in this paper, [12], and [19] for Case 2.

**Figure 8:**
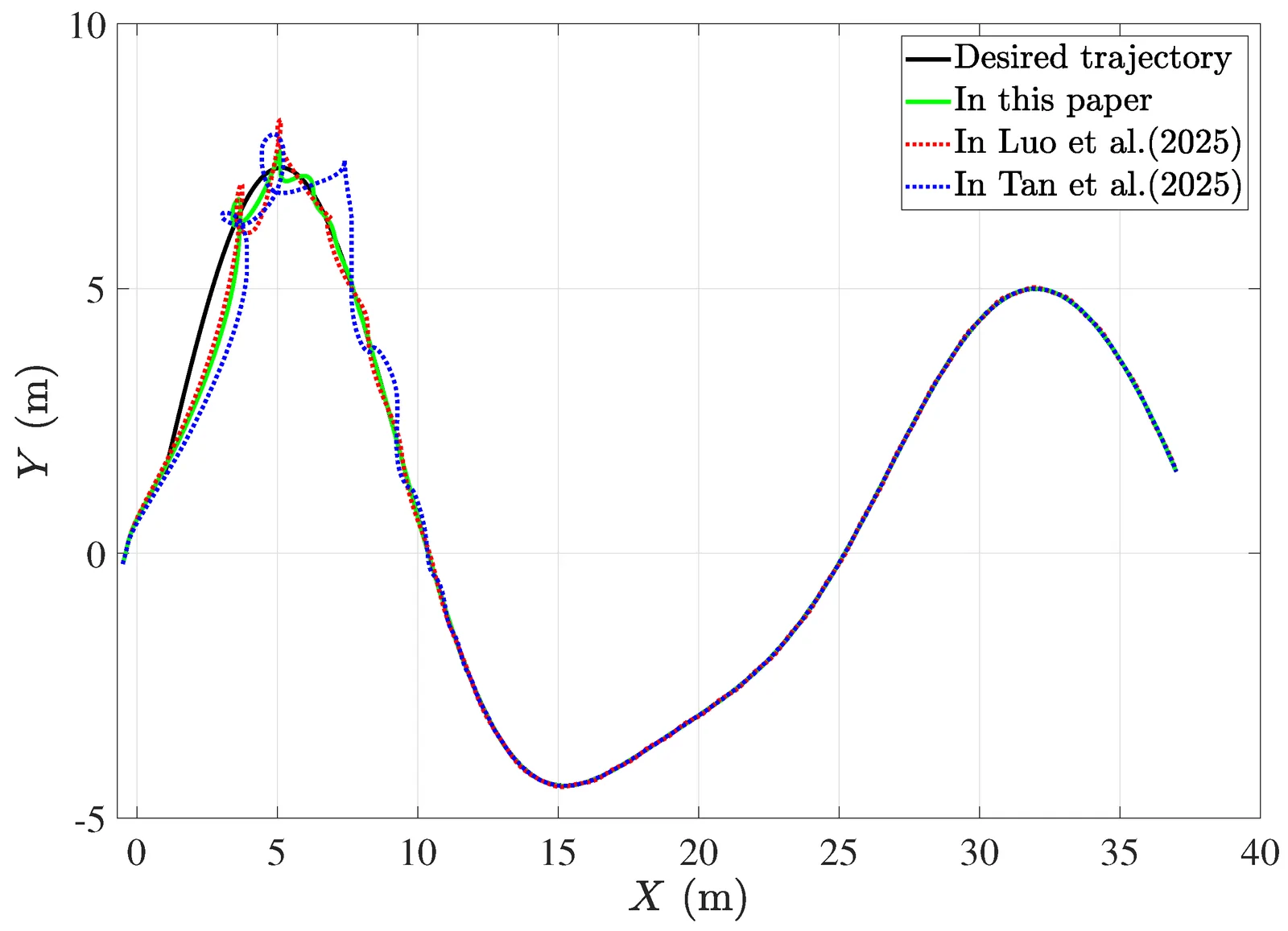
The tracking trajectories of the three methods in this paper, [12], and [19] for Case 3.

**Figure 9:**
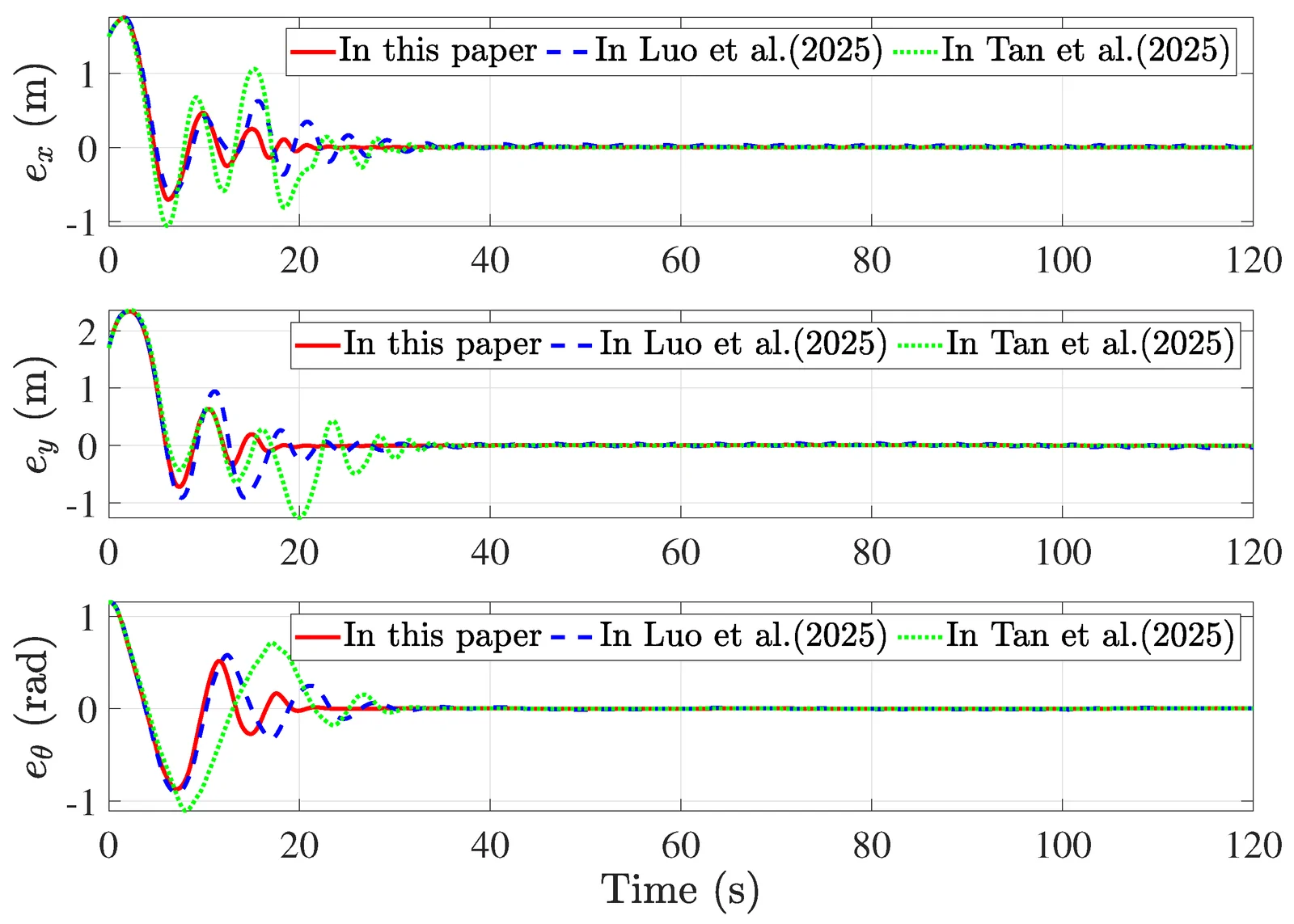
The trajectories of tracking errors in this paper, [12], and [19] for Case 3.

**Table 1:** Tracking accuracy comparison using RMSE for Case 1.

RMSE	$e_{x}$	$e_{y}$	$e_{\theta }$
In this paper	0.061 m	0.068 m	0.107 rad
In [13]	0.134 m	0.111 m	0.112 rad
In [19]	0.078 m	0.081m	0.113 rad

**Table 2:** Number of triggers of different methods for Case 1.

Number of Triggers	$\tau_{s1}$	$\tau_{s2}$	$\tau_{s3}$	$\tau_{s4}$
In this paper	660	617	665	604
In [13]	940	953	938	976
In [19]	2406	2477	2668	3264

**Table 3:** Tracking accuracy comparison using RMSE for Case 2.

RMSE	$e_{x}$	$e_{y}$	$e_{\theta }$
In this paper	0.050 m	0.033 m	0.091 rad
In [13]	0.079 m	0.037 m	0.146 rad
In [19]	0.059 m	0.040 m	0.097 rad

**Table 4:** Number of triggers of different methods for Case 2.

Number of Triggers	$\tau_{s1}$	$\tau_{s2}$	$\tau_{s3}$	$\tau_{s4}$
In this paper	223	241	233	235
In [13]	570	524	562	528
In [19]	645	516	584	497

**Table 5:** Tracking accuracy comparison using RMSE for Case 3.

RMSE	$e_{x}$	$e_{y}$	$e_{\theta }$
In this paper	0.296 m	0.438 m	0.208 rad
In [13]	0.311 m	0.471 m	0.226 rad
In [19]	0.356 m	0.488 m	0.272 rad

**Table 6:** Number of triggers of different methods for Case 3.

Number of Triggers	$\tau_{s1}$	$\tau_{s2}$	$\tau_{s3}$	$\tau_{s4}$
In this paper	323	397	312	389
In [13]	411	444	394	449
In [19]	1113	942	1103	947

## Data Availability

The original contributions presented in this study are included in the article. Further inquiries can be directed to the corresponding author.
